# Rasch Validation of the Arabic Version of the Chedoke–McMaster Attitudes Toward Children With Handicaps (CATCH-AR) Scale

**DOI:** 10.3389/fpsyg.2019.02924

**Published:** 2020-01-24

**Authors:** Ghaleb H. Alnahdi

**Affiliations:** Special Education Department, College of Education, Prince Sattam Bin Abdulaziz University, Al-Kharj, Saudi Arabia

**Keywords:** CATCH, psychometric properties, attitudes, peers with disability, inclusive education

## Abstract

Students’ attitudes toward peers with disabilities are crucial for the social inclusion of the latter. Therefore, understanding such attitudes can help improve the social inclusion of students with disabilities. This study aimed to examine the psychometric properties of the Arabic version of the Chedoke–McMaster Attitudes toward Children with Handicaps scale. Data were collected from 415 elementary school students, including 232 (56%) girls and 183 (44%) boys, in grades three to six in Saudi Arabia. The psychometric properties of the scale were examined using the Rasch analysis procedures. The results did not support the unidimensionality of the 36-item scale. Dividing items based on whether they are negatively or positively phrased improved the scale fit. Both the 15-item (positive phrasing) and the 18-item (negative phrasing) scales were supported by the Rasch analysis as unidimensional scales.

## Introduction

In recent years, inclusive education practices around the world have started to develop. This shift can help to increase the opportunities of children with disabilities to experience more contact with their peers without disabilities. Nevertheless, improving inclusive practices to increase peer contact is not always a straightforward process, and there is no guarantee that children with disabilities will have successful social experiences (Rosenbaum et al., 1986; Bossaert and Petry, 2013). One of the main ingredients for successful peer inclusion is the attitudes of children toward their peers with disabilities (McDougall et al., 2004). Simply being physically in school is not the goal of inclusion, and positive peer attitudes play a central role in helping children with disabilities to adapt to their new environment (Scior et al., 2013). In addition, gaining a better understanding of attitudes toward people with disabilities could be informative in predicting behavior toward this population (Ajzen and Fishbein, 1980; Kraus, 1995).

When viewed in this context, examining students’ attitudes toward peers with disabilities becomes crucial. Yet, despite the importance of this issue, finding a reliable scale to assess such attitudes is not always an easy task. This can be especially difficult when assessing Arabic-speaking populations, as the current number of scales with Arabic-language versions is limited. One of the scales most commonly used for this purpose is the Chedoke–McMaster Attitudes towards Children with Handicaps (CATCH) scale (Rosenbaum et al., 1986). This scale was developed to measure children’s attitudes toward peers with disabilities and has shown acceptable psychometric properties with various sample populations (Rosenbaum et al., 1986, 1988; Vignes et al., 2008; Armstrong et al., 2017). The CATCH scale covers three components regarding the attitudes of children toward peers with disabilities, and is viewed as one of the best scales for this purpose (Vignes et al., 2008). The CATCH scale was conceptually developed based on the three-factor attitudes model proposed by Triandis in 1971 (Rosenbaum et al., 1986); thus, it comprises three subscales: (A) affective attitudes (“I would be afraid of a handicapped child”); (B) behavioral intention (“I would talk to a handicapped child I didn’t know”); and (C) cognitive attitudes (“Handicapped children are as happy as I am”). The scale was developed to measure children’s attitudes; however, it has been used with students as old as 16 (Bossaert and Petry, 2013). Furthermore, the CATCH scale validity structure has been examined in previous studies. Rosenbaum et al. (1986) found that the data supported a model with two factors instead of three, with the affective and behavioral factors grouped together in one subscale and the cognitive factor serving as the other. However, Armstrong et al. (2017) concluded that the three subscales should continue to be used separately, as the combined subscales would not provide a unidimensional variable.

In other studies, the cognitive attitudes subscale (C) proved to be problematic. For example, in a study performed in Netherlands, the cognitive subscale was found to negatively influence the data fit (De Boer et al., 2012). Additionally, a study conducted in England using the Rasch model concluded that the cognitive subscale should be used cautiously, as it is not a unidimensional subscale and has a low internal consistency (Armstrong et al., 2017). As for the Arabic version of the CATCH scale (CATCH-AR), a previous study examined its construct validity using confirmatory factor analysis and found that the hypothesized three-factor structure of the 36-item CATCH-AR was not supported (Alnahdi, 2020). It was found that negatively phrased items were perceived differently by the participants compared with positively phrased items. A statement is considered to be positively phrased when agreeing with it would indicate a positive attitude. For example, Item 9, “I would invite a handicapped child to my birthday party,” is a positively phrased statement. A statement is considered negatively phrased when disagreeing with it would indicate a positive attitude. For example, item 12, “Handicapped children don’t like to make friends,” is a negatively phrased statement. Recent research also found that by removing the negatively phrased items, the data showed an improved model fit. Cronbach’s alpha was 0.843 for the whole scale, and 0.636, 0.651, and 0.542 for subscales A, B, and C, respectively; for the positive 18-item scale, it was 0.861, and for the negative 18-item scale, 0.772 (Alnahdi, 2020).

Since the 36-item CATCH-AR scale did not show its hypothesized three-factor structure without the removal of negatively phrased items (Alnahdi, 2020), our study aimed to further investigate the CATCH-AR scale factor structure by using a Rasch analysis with a sample population living in Saudi Arabia. We feel this could contribute to the literature, as most previous studies have used the classical test theory approach, and only one other study has used the Rasch analysis (Armstrong et al., 2017), and their findings did not support the unidimensionality of the 36 items scale. We feel that applying the Rasch analysis to the CATCH-AR scale could help us to better understand whether this is a unidimensional scale that can be used to combine the scores of all 36 items to produce a total score that indicates children’s attitudes toward peers with disabilities. Alternatively, we can learn if the scale is not, in fact, unidimensional, but instead has a multidimensional structure, with scores to be calculated at the subscale level only.

We aimed to address two research questions in this study. First, we examined whether the 36-item CATCH-AR fits the Rasch model as a unidimensional scale. Second, we explored if the total sum score of the 36-item CATCH-AR can be used to accurately represent children’s attitudes toward their peers with disabilities.

## Materials and Methods

The sample population of this study comprised 415 elementary school students in Saudi Arabia. Of this sample, 232 (56%) were female and 183 (56%) male, with an age range of 9–11 years. This study was approved by the Institutional Review Board at Prince Sattam Bin Abdulaziz University. Additionally, consent from the students’ families was obtained by the school administrators before the study was conducted.

The Arabic version of the scale CATCH-AR (Alnahdi, 2019, 2020) used in this study. We gave participants four options for rating the scale items in this study. The score codes for positively phrased items were as follows: Strongly Agree = 3, Agree = 2, Disagree = 1, and Strongly Disagree = 0. For negatively phrased items the codes were reversed: Strongly Agree = 0, Agree = 1, Disagree = 2, and Strongly Disagree = 3. Since the coding was reversed for the negatively phrased items so all were substantively coded similarly. Therefore, higher total scores indicated more positive attitudes toward peers with disabilities.

In order to conduct the Rasch analysis, we used the Rasch Unidimensional Measurement Model (RUMM2030) software (Andrich et al., 2010) and followed the guidelines recommended by Tennant and Conaghan (2007). We considered the overall fit to be good if there was a non-significant chi-squared distribution for the item-trait interaction and if the residual means of the total item and person scores were around zero, with a standard deviation around 1 (Alnahdi, 2018). To identify any unacceptable responses, we reviewed the disorder threshold via comparison of the threshold map and the item characteristic curve (ICC). “For a well-fitting item you would expect that, across the whole range of the trait being measured, each response option would systematically take turns showing the highest probability of endorsement” (Pallant and Tennant, 2007, p. 6).

We verified item fit by identifying any residual items within an acceptable ±2.5 range that displayed a statistically significant difference from other items within that range (Tennant and Conaghan, 2007). We checked for local item dependence (LID) by reviewing our data for high correlations between item residuals after extracting the latent variable (attitudes). We considered an item to be a violation of the Rasch model’s local dependency assumption if we found a value of 0.30 above the average of the residual correlations (Christensen et al., 2017). We examined the unidimensionality of the scale using the RUMM2030 software, following the guidelines set by Tennant and Conaghan (2007) for Smith’s test of unidimensionality (Smith, 2002). We then conducted a principal component analysis (PCA) of the residuals. Next, we created two ability estimates for each person in the sample. The first ability estimate was derived from items with positive loadings on the first PCA component, while the second ability estimate was derived from items with negative loadings.

We then conducted *t*-tests to examine whether the two ability estimates showed a statistically significant difference. *t*-tests showing a statistical significance should not exceed 5% of the sample or the lower limit of 95% for the binomial proportion confidence intervals at 5% level or less (Smith, 2002; Tennant and Conaghan, 2007; Hadzibajramovic et al., 2015; Alnahdi, 2018). Internal consistency was verified by identifying a value of 0.7 on the person separation index (PSI) (Tennant and Conaghan, 2007). Finally, we created a transformation table displaying the raw and interval scores and used the comparisons it provided to better understand changes in attitudes of children toward peers with disabilities. As with interval scores, the distance between all scores are equal. We used the following formula for this transformation: *Y* = *M* + (*S* × logit score), where *S* = range of interval-level scale (60 for a 0–60 scale) divided by the actual range of logit scores, and *M* = (minimum score of interval-level scale) – (minimum logit score × *S*) (Alnahdi, 2018, p. 355).

## Results

We first examined the 36-item CATCH-AR scale to determine its fit to the Rasch model. The results (Table 1) showed that the complete 36-item CATCH-AR scale was not unidimensional. For the 36-item CATCH-AR scale, 35% of the *t*-tests used to identify unidimensionality were significant, compared to the ideal percentage of 5%. In our second test, we removed 35 participants who did not fit the standards of the analysis, which reduced our sample size to 380. However, the 36-item CATCH-AR scale was still not found to be unidimensional, as the percentage of significant *t*-tests was still nowhere close to the required 5%, but instead remained at 35%. As both tests showed similar results, we determined that it is not appropriate to use the 36-item CATCH-AR scale to provide unidimensional measurements.

**TABLE 1:** Rasch statistics for each tested solution for the Arabic version of Chedoke–McMaster Attitudes toward Children with Handicaps scale.

		**Itemresidual fit**		**Person residual fit**		**Item-trait interaction**			**Unidimensionality *t*-tests**	
					
**Solutions for a unidimensional scale**	**Scale**	**Mean**	***SD***	**Mean**	***SD***	***x*^2^ (df)**	***p***	**PSI**	**% of significant tests**	**Lower limit of 95% CI**
	**36-item scale**	**0.801**	**1.61**	**−0.18**	**0.387**	**817.04 (216)**	**0.000**	**0.797**	**35.66**	**16.7**
	**36-item scale (removed 35 misfit participants)**	**0.661**	**1.58**	**−0.22**	**2.040**	**758.32 (216)**	**0.000**	**0.793**	**35.00**	**30.2**
Separate subscales: Armstrong et al. (2017)	A (12 items)	1.152	1.99	−0.28	1.629	275.93 (72)	0.000	0.590	14.46	11.2
	B (12 items)	1.295	1.82	−0.26	1.628	180.60 (72)	0.000	0.608	19.52	15.8
	C (12 items)	0.523	1.47	−0.34	1.489	258.53 (72)	0.000	0.581	10.36	7.60
Two subscales together (A + B): Rosenbaum et al. (1986)	A + B (24 items)	1.104	1.89	−0.23	0.437	595.13 (144)	0.000	0.754	30.60	26.2
Item divided based on phrasing direction: Alnahdi (2020)	18-item (positively phrased)	0.170	1.99	−0.113	1.052	213.41 (108)	0.000	0.795	9.74	6.94
	15-item (positively phrased; 3 misfit items were removed: 3, 17, 19)	0.767	1.88	−0.269	1.470	166.04 (90)	0.000	0.789	7.63	5.17
	15-item (items were rescored)	0.201	1.75	−0.146	1.021	148.03 (90)	0.000*	0.773	**5.00**	**3.03**
	18-item (negatively phrased)	0.843	1.595	−0.247	1.698	252.2 (108)	0.000	0.793	11.32	8.31
	18-item (negatively phrased) (items were rescored)	−0.323	1.404	−0.234	1.071	189.15 (90)	0.000*	0.729	**5.00**	**3.03**
Ideal values		0.0	<1.4	0.0	<1.4		>0.05	>0.7	≤5	≤5

CI, confidence interval in the binomial test of proportions; df, degrees of freedom; PSI, person separation index; SD, standard deviation; A, affective attitude subscale; B, behavioral intention subscale; C, cognitive attitude subscale, +, positively worded items only; -, negatively worded items only. *By adjusting the sample size to 200, *p* > 0.05 and non-significant chi-squared as a good indicator of model fit; Bold font, good indicator of unidimensionality.

We next looked at other solutions, based on previous studies. In an attempt to find a solution that supported a unidimensional scale, we ran a separate analysis for each subscale, as recommended by Armstrong et al. (2017). The subscales each consisted of 12 items. None of the results from these tests supported any of the three subscales to be used as a unidimensional scale. Next, we looked at the two-factor solution, with A and B subscales combined and C as a separate subscale, as Rosenbaum et al. (1986) tested by confirmatory factor analysis. However, the cognitive subscale (C) was excluded, as its psychometric properties were not supported in the study by Armstrong et al. (2017). In this test of the 24-item scale (A and B subscales combined), the results did not support this scale as an appropriate unidimensional measure (Table 1).

Finally, we reviewed the CATCH-AR scale with items separated by negative and positive phrasing, as previously suggested by Alnahdi (2020). Two tests were run, one with positively phrased items and one with negatively phrased items. Each group of items was an 18-item scale with three 6-item subscales. The results for the scale with positively phrased items showed that this 18-item scale was close to being a unidimensional scale. However, three items from the cognitive subscale (Items 3, 17, and 19) were significantly misfit, with fit residuals at +2.5. Therefore, these three items were removed. We then tested the resulting 15-item scale and found that the results supported its use as a unidimensional scale. In addition, its internal consistency was acceptable with a PSI > 0.7 (0.773). Furthermore, we obtained similar results supporting the unidimensionality of the 18-item scale with negatively phrased items, which also showed acceptable internal consistency with a PSI of 0.729.

**FIGURE 1:**
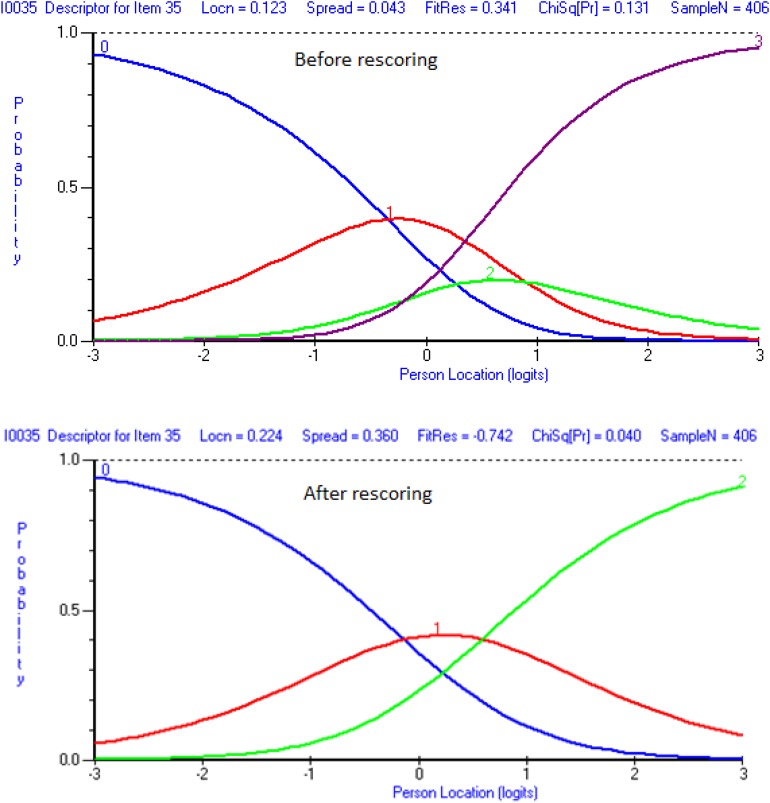
Item 35 before and after rescoring.

Threshold maps were then reviewed for both scales to ensure there was no threshold disorder for any items. However, as shown in Figure 1, all items showed threshold disorder (upper chart in Figure 1) except for item 19. Therefore, we re-scored items by combining adjacent categories (lower chart in Figure 1), as the recommended solution indicates (Tennant and Conaghan, 2007). Table 2 shows the new scores for the 15-item scale. A similar procedure was also conducted for the 18-item scale, and the new scores are displayed in Table 3.

**TABLE 2:** 15-Item Arabic version of the Chedoke–McMaster Attitudes toward Children with Handicaps (CATCH-AR) scale with new scores (positively phrased items).

**Item**		**Strongly disagree**	**Disagree**	**Agree**	**Strongly agree**	**Domain**
1	Item 1	0	1	2	2	A
2	Item 5	0	1	2	2	C
3	Item 7	0	0	1	1	B
4	Item 9	0	1	2	2	B
5	Item 11	0	1	2	2	B
6	Item 13	0	1	2	2	A
7	Item 15	0	1	2	2	A
8	Item 21	0	0	1	1	A
9	Item 23	0	0	1	1	A
10	Item 25	0	0	1	1	B
11	Item 27	0	1	2	2	C
12	Item 29	0	0	1	1	B
13	Item 31	0	1	2	2	A
14	Item 33	0	1	2	2	C
15	Item 35	0	1	2	2	B

*A, affective attitude; B, behavioral intention; C, cognitive attitude.*

**TABLE 3:** 18-Item Arabic version of the Chedoke–McMaster Attitudes toward Children with Handicaps (CATCH-AR) scale with new scores (negatively phrased items).

**Item**		**Strongly disagree**	**Disagree**	**Agree**	**Strongly agree**	**Domain**
1	Item 2	0	0	1	1	B
2	Item 4	0	0	1	1	B
3	Item 6	0	0	1	1	A
4	Item 8	0	0	1	1	C
5	Item 10	0	0	1	1	A
6	Item 12	0	0	1	1	C
7	Item 14	0	0	1	1	C
8	Item 16	0	0	1	1	B
9	Item 18	0	0	1	1	A
10	Item 20	0	0	1	1	B
11	Item 22	0	0	1	1	B
12	Item 24	0	0	1	1	C
13	Item 26	0	0	1	1	A
14	Item 28	0	0	1	1	A
15	Item 30	0	0	1	1	C
16	Item 32	0	0	1	1	B
17	Item 34	0	0	1	1	A
18	Item 36	0	0	1	1	C

*A, affective attitude; B, behavioral intention; C, cognitive attitude.*

Next, differential item functioning (DIF) analyses were conducted to ensure that items in the 15- and 18-item scales performed similarly for boys and girls. For the 15-item scale, one item (Item 27) displayed DIF when separated by gender. This item reads “Handicapped children are interested in lots of things.” To ensure the DIF displayed by this item did not negatively influence the person parameters, we conducted two calibrations. We performed one calibration of the 15-item scale including Item 27, and then another calibration with a 14-item scale after removing Item 27. By comparing the location of all the samples from these two calibrations, we found only five cases (1.3% of the sample) where the person location changed by more than 0.5 logit. Since this percentage was lower than the acceptable criteria of 5% of the sample, we assumed there was no impact from these two items on the person parameters (Tennant and Pallant, 2007), and kept Item 27 in the 15-item scale. For the 18-item scale, one item (Item 2) displayed DIF by gender. This item reads “I wouldn’t introduce a handicapped child to my friend.” We followed a similar procedure to ensure that the DIF displayed by this item did not negatively influence the person parameters. In this case, less than 0.3% of the sample showed a change in location larger than 0.5 logit, which was also below the recommended criteria of 5%. Therefore, no further action needed to be taken. Table 4 shows the item statistics for the 15-item scale, and Table 5 shows the item statistics for the 18-item scale.

**TABLE 4:** Item fit statistics for 15-item Arabic version of the Chedoke–McMaster Attitudes toward Children with Handicaps (CATCH-AR) scale.

**Item**	**Location**	**SE**	**Fit residual**	**χ^2^**	***p*^a^**
7	2.302	0.221	–0.052	5.098	0.5313
23	0.873	0.142	0.718	9.852	0.1310
5	0.549	0.091	1.082	13.215	0.0397
21	0.29	0.126	2.848	10.96	0.0896
33	0.051	0.082	0.673	5.971	0.4264
1	–0.034	0.082	–1.44	12.314	0.0553
35	–0.11	0.081	–0.653	8.254	0.2201
9	–0.171	0.079	0.763	5.908	0.4336
13	–0.248	0.082	–0.343	7.31	0.2931
25	–0.377	0.117	–2.646	15.864	0.0145
15	–0.386	0.079	–1.775	11.914	0.0639
31	–0.442	0.079	–2.207	13.124	0.0411
27	–0.556	0.078	3.41	17.151	0.0087
11	–0.635	0.076	1.575	8.035	0.2355
29	–1.106	0.117	1.074	3.062	0.8010

*SE, standard error. ^*a*^Bonferroni adjusted *p* = 0.0033 (0.05/15).*

**TABLE 5:** Item fit statistics for 18-item Arabic version of the Chedoke–McMaster Attitudes toward Children with Handicaps (CATCH-AR) scale.

**Item**	**Location**	**SE**	**Fit residual**	**χ^2^**	***p*^a^**
26	1.093	0.121	–2.602	7.116	0.212
10	0.977	0.12	–0.642	4.647	0.460
24	0.954	0.119	–1.509	5.08	0.406
16	0.876	0.119	–2.11	11.63	0.040
12	0.709	0.117	–0.98	7.671	0.175
22	0.629	0.116	–0.603	3.71	0.592
20	0.575	0.116	–1.875	6.479	0.262
18	0.358	0.115	–1.004	7.835	0.166
32	0.323	0.115	–1.595	4.919	0.426
28	0.256	0.115	–0.457	3.333	0.649
30	0.125	0.115	–0.767	3.73	0.589
2	0.052	0.115	1.482	3.077	0.688
14	0.044	0.115	1.138	6.798	0.236
4	–0.353	0.117	2.275	5.833	0.323
34	–1.017	0.127	0.529	6.436	0.266
36	–1.793	0.152	0.343	4.544	0.474
6	–1.826	0.153	1.29	12.037	0.034
8	–1.982	0.16	1.257	21.225	0.001

*SE, standard error. ^*a*^Bonferroni adjusted *p* = 0.00055 (0.05/18).*

To sum up, the data from the 36-item CATCH-AR did not fit the Rasch model. Moreover, the study findings did not support the unidimensionality of a scale including the A and B subscales only. However, by separating items based on phrasing (negatively or positively phrased), the fit of the resulting scales to the Rasch model did improve. Both the 15-item scale with positively phrased items and the 18-item scale with all negatively phrased items were supported as unidimensional scales.

Before this step, the item statistics for the 15-item scale and the new scores for all items were computed. However, the distance between any two raw scores resulting from the Rasch calibration is still difficult to interpret. Therefore, raw scores were transformed into interval scores to ensure that any improvement in one unit would have equal weight across the entire scale (Table 6). A similar procedure was conducted to transform raw scores for the 18-item scale into interval scores (Table 7).

**TABLE 6:** Transformation table for 15-item Arabic version of the Chedoke–McMaster Attitudes toward Children with Handicaps (CATCH-AR) scale.

**Row score**	**Interval score**	**Row score**	**Interval score**	**Row score**	**Interval score**
0	0.00	9	23.97	18	35.31
1	6.55	10	25.20	19	36.93
2	10.97	11	26.41	20	38.74
3	13.95	12	27.59	21	40.88
4	16.26	13	28.78	22	43.51
5	18.16	14	29.97	23	46.99
6	19.81	15	31.21	24	52.20
7	21.30	16	32.48	25	60.00
8	22.67	17	33.85		

**TABLE 7:** Transformation table for 18-item Arabic version of the Chedoke–McMaster Attitudes toward Children with Handicaps (CATCH-AR) scale.

**Row score**	**Interval score**	**Row score**	**Interval score**	**Row score**	**Interval score**
0	0.00	7	27.33	14	42.37
1	7.20	8	29.49	15	45.15
2	12.53	9	31.57	16	48.55
3	16.47	10	33.60	17	53.32
4	19.71	11	35.64	18	60.00
5	22.52	12	37.74		
6	25.03	13	39.95		

## Discussion

Our results showed that the data from the 36-item CATCH-AR scale was not unidimensional and did not fit the Rasch model. This result is consistent with the findings of Armstrong et al. (2017), which indicated that the 36-item CATCH scale was not supported for use as a unidimensional tool to assess children’ attitudes toward peers with disabilities.

Additionally, our findings did not support the unidimensionality of the affective (A) and behavioral intention (B) subscales when combined together into a 24-item scale. This was examined to determine if we could support the findings of Rosenbaum et al. (1986) indicating that the A and B subscales could be combined as one factor (one latent variable). However, our results did not support the unidimensionality of this 24-item scale. In addition, our findings did not support the recommendation of Armstrong et al. (2017) to use the A, B, and C subscales as three separate scales. However, we did find a good fit for the three subscales together when their items were separated by phrasing. This indicates that a total score of these items combined can be used as an indicator of children’s attitudes toward peers with disabilities. This finding is also more supportive of the use of the cognitive subscale (C) than was the study by Armstrong et al. (2017), which suggested that researchers should use the cognitive subscale with caution, as it did not behave as a unidimensional and internally consistent scale.

Our study found that children perceived items differently based on whether they are phrased positively or negatively. The Rasch analysis for the 36-item CATCH-AR scale containing 18 positively phrased items and 18 negatively phrased items was not successful. Furthermore, for the 24-item scale with the A and B subscales combined, as was proposed by Rosenbaum et al. (1986), around 30% (ideal is 5%) of the *t*-tests run to determine unidimensionality were significant. When separating these 24 items into two 12-item subscales based on phrasing, the percentage decreased significantly to 7 and 5%, respectively. These findings may indicate that item phrasing is a dimension on its own, which makes it difficult to fit the data to a unidimensional Rasch model with phrasing contributing to the effect.

We examined this effect by dividing the subscales based on negative or positive phrasing, as suggested by Alnahdi (2020). The results showed that having negatively and positively phrased items combined did influence the fit of the data to the Rasch model. Therefore, this could also cause a similar unwanted influence with other samples if researchers try to separate items based on phrasing. For example, Armstrong et al. (2017) made the following changes to a few negatively phrased items to improve the fit of the scale: Item 2 was rephrased from “I would not introduce a handicapped child to my friends” to “I would introduce a disabled person to my friends;” Item 20 was rephrased from “In class I wouldn’t sit next to a handicapped child” to “I would sit next to a disabled person;” and Item 32 was rephrased from “I would not go to a handicapped child’s house to play” to “I would go to a disabled person’s house if I was invited.”

The influence of having both positive and negative phrasing combined has been documented in different studies. For instance, it was found that negatively phrased items performed differently compared with other items (Stewart and Frye, 2004). Benson and Hocevar (1985) found that using mixed phrasing styles may negatively influence the validity of attitude measures, a finding that has been supported by other research recommending the use of direct wording (Barnette, 2000; Stewart and Frye, 2004). Furthermore, Salazar (2015, p. 192) found that using both positively and negatively phrased items “seriously affected the internal consistency of the scales.”

This should be considered when developing new scales. In particular, the use of phrases in only one direction should be considered, especially with children, as the process of reading items in different directions require more cognitive effort and more time for each item. Normally, students are likely to assume an item to be in the same direction as previous items, particularly if only a few seconds are given per item. As a result of such cases, inconsistencies between the responses to the positively phrased items and other statements are expected.

Finally, the raw scores for both scales were converted into interval scores. This step is important for both researches and users of the scales, since it makes it easier to interpret the obtained scores. For example, by having raw scores only, an improvement in the score from 14 to 15 might be considered equal to an improvement from 23 to 24. However, by being able to review interval scores, we know that the improvement from 23 to 24 is 5.21 as an interval score (52.20 - 46.99 = 5.21), while the improvement from 14 to 15 is 1.24 as an interval score (31.21 - 29.97 = 1.24). This means that the improvement from 23 to 24 is about four times greater than that from 14 to 15. This could be very helpful in understanding the effectiveness of intervention programs designed to improve children’s attitudes toward peers with disabilities.

## Conclusion

Based on our study findings, we recommend that researchers using the Arabic-translated CATCH-AR scale use the 15-item or 18-item version of the scale to measure children’s attitudes toward peers with disabilities. Calculating a total score from the 36-item CATCH-AR scale was not supported by the findings of this study. Calculating a total score from the 15-item or 18-item versions of the CATCH-AR scale was, however, supported by our Rasch analysis to obtain a unidimensional measurement of students’ attitudes toward peers with disabilities. Furthermore, researchers who do want to use the complete 36-item CATCH-AR scale are recommended not to calculate the total score by summing all item scores. Additionally, researchers who want to use only one subscale from the CATCH-AR can treat any one of the three subscales in the 15-item CATCH-AR as unidimensional: the 6-item subscale A, 6-item subscale B, and 4-item subscale C. Moreover, for researchers who would like to include the items that were removed from the 36-item CATCH-AR, we recommend to rephrase them in a way that agreeing with any of the statements would indicate having a positive attitude toward peers with disabilities.

## Data Availability Statement

The datasets generated for this manuscript are not publicly available. Requests to access the datasets should be directed to GA, ghalnahdi@gmail.com and g.alnahdi@psau.edu.sa.

## Ethics Statement

The studies involving human participants were reviewed and approved by Prince Sattam Bin Abdulaziz University IRB. Written informed consent to participate in this study was provided by the participants’ legal guardian/next of kin.

## Author Contributions

GA drafted and analyzed the manuscript.

## Conflict of Interest

The author declares that the research was conducted in the absence of any commercial or financial relationships that could be construed as a potential conflict of interest.
